# Stump Appendicitis: An Uncompleted Surgery, a Rare but Important Entity with Potential Problems

**DOI:** 10.1155/2013/972596

**Published:** 2013-04-04

**Authors:** J. A. A. Awe, A. M. Soliman, R. W. Gourdie

**Affiliations:** Northern Area Armed Forces Hospital, King Khalid Military City, Hafr Al-Batin 31991, Saudi Arabia

## Abstract

Appendicectomy for appendicitis is one of the commonest surgical procedures performed worldwide. The residual appendiceal stump left after an initial appendectomy risks the development of stump appendicitis. Stump appendicitis is a real recognized entity but not often considered when evaluating patients with right lower quadrant abdominal pain, especially those with past history of appendectomy. It remains a clinical challenge with the result that its diagnosis and effective treatment are often delayed with possible attendant morbidity or mortality. Stump appendicitis results from obstruction of the lumen of the remaining appendix stump, usually by a faecolith. This increases intraluminal pressure, impairing venous drainage and allowing subsequent bacterial infection. We present the case of a twenty-five (25)-year-old female who underwent laparoscopic appendicectomy and presented four and half (4(1/2)) months later with fever, right lower quadrant abdominal pain, and tenderness associated with repeated vomiting. Exploratory laparotomy was carried out after clinical and imaging studies which revealed big inflammatory mass with abscess at the right iliac fossa and recurrent appendicitis of the appendiceal stump. Surgical treatment is easy but recognition of this important entity but potentially dangerous condition should always be borne in mind in order to avoid delay in its diagnosis and treatment.

## 1. Introduction

Acute Appendicitis is one of the most common causes of abdominal pain, and it is one of the common surgical emergencies treated by general surgeons.

Most surgical personnel are quite familiar with the common complications after appendectomy such as wound infections and pelvic abscesses [1].

The postoperative development of stump appendicitis is an exceedingly rare entity with only 36 reported cases in the English language literature [2–4]. Stump appendicitis is an acute inflammation of the residual appendiceal stump and is an underreported complication that can occur after open or laparoscopic appendectomy [5–13].

The entity has been recorded as occurring from about three weeks to an interval of twenty-three (23) years after appendectomy, but our case presented only four and half (4(1/2)) months after laparoscopic appendectomy [14, 15].

With the introduction of laparoscopic appendectomy in the last fifteen to twenty (15–20) years; however, incidence of stump appendicitis has probably increased even though this was not supported by the findings of Liang et al. [3].

The fact that the diagnosis of stump appendicitis is usually not considered as the possible etiology for right lower quadrant abdominal pain in patients with prior appendectomy creates a delay in making the correct diagnosis and explains why the rate of perforation for stump appendicitis approaches 70% [16, 17].

We present this case of a twenty-five (25)-year-old female who presented in our emergency room (ER) department with right iliac fossa abdominal pain and repeated vomiting four and half (4(1/2)) months after laparoscopic appendectomy.

It is to draw attention to the fact that stump appendicitis with all its attendant complications such as perforation, abscess formation is real and should be borne in mind in the differential diagnosis of patients presented with right iliac fossa pain after appendectomy [18].

## 2. Case Report

A twenty-five (25)-year-old lady presented with a two-day history of stabbing abdominal pain located first in the epigastric area but later migrated to the right lower abdomen with associated repeated vomiting. This pain became more intense and continuous in the right lower abdomen.

Her last menstrual period was one week prior to presentation at the emergency room (ER).

Significant in her past medical history is the fact that she underwent laparoscopic appendectomy four and half (4(1/2)) months earlier.

On admission, the patient looked unwell with a fever of 38°C, BP = 90/60 mm Hg, and Pulse = 92/min RR = 28/min. Abdomen was tender all over but maximum in the right iliac fossa with guarding and rigidity. Bowel sounds were sluggish.

Plain abdominal X-ray and urinalysis were normal, but the total white cell count (WBC) was 19000 with 85% neutrophils.

**Figure 1:**
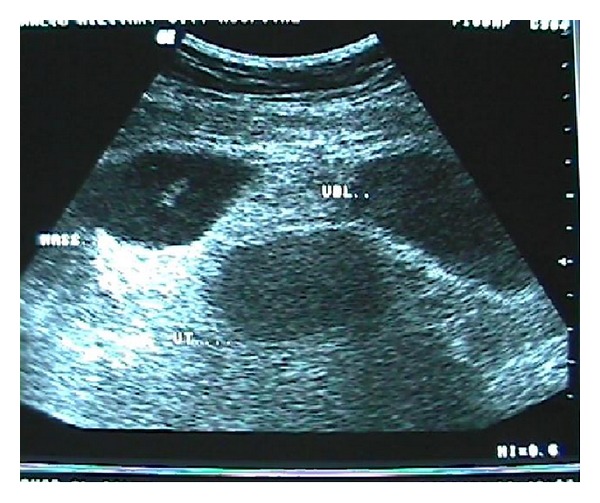


**Figure 2:**
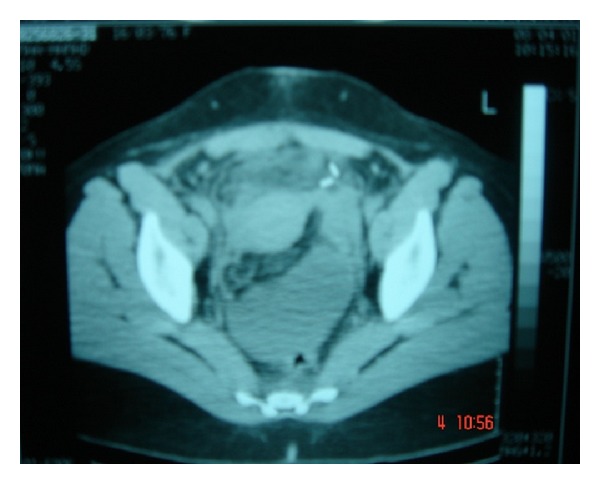


Abdominal ultrasound (U/S) (Figure 1) and abdominal computerized tomographic scan (CT) (Figure 2) revealed inflammatory, intraperitoneal collection at the right iliac fossa (RIF) which was diagnosed radiologically as an inflammatory mass with abscess.

IV fluid with parenteral antibiotics was commenced.

The patient underwent exploratory laparotomy through lower midline incision with a view to draining the abscess.

**Figure 3:**
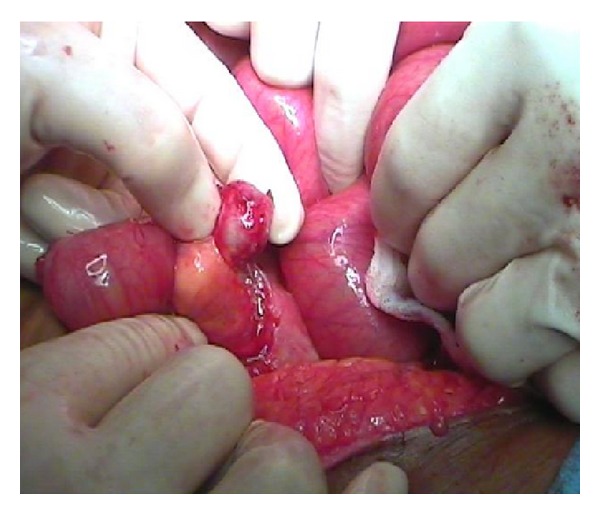
Residual appendix.

**Figure 4:**
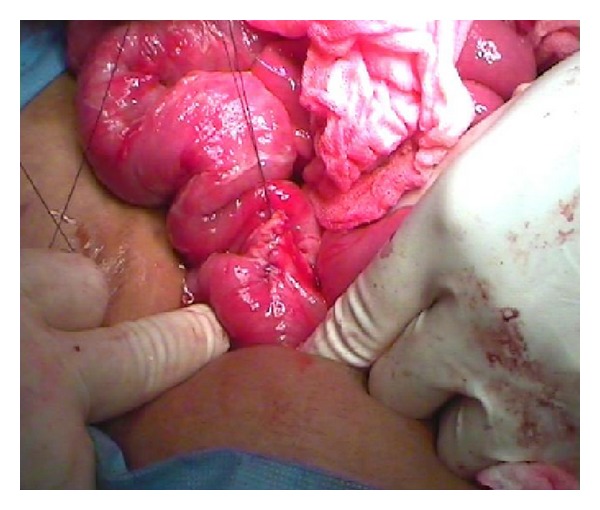
Suturing of base.

At laparotomy, a significant stump of the original appendix was left behind, inflamed with missed faecolith at the base of the appendix. It was perforated with abscess formation (Figures 3 and 4).

The appendiceal stump with the left-behind faecolith was ligated, abscess drained followed by peritoneal toilet and wound closed in layers with a drain left in situ.

**Figure 5:**
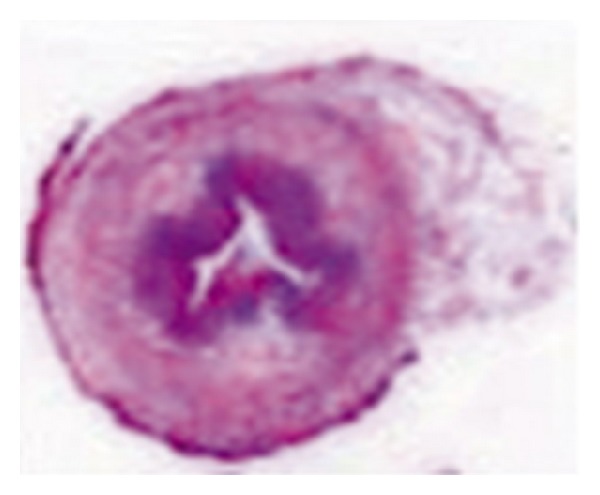
Slide of appendix.

Histopathological examination of the ligated appendix stump confirmed the presence of an appendix with inflammation of surrounding adjacent tissue and abscess (Figure 5).

Postoperatively, she did well and was discharged home after one week and has since been discharged from surgical outpatient followup.

## 3. Discussion and Conclusion

Baumgardner in 1949 [15] was the first to describe stump appendicitis, and since then a total of 36 cases have been reported in a comprehensive review of the English language literature [2, 3].

Some reports have suggested that laparoscopic appendectomy is associated with an increased incidence of stump appendicitis when compared with open appendectomy.

However, the most recent comprehensive review of the literature examining thirty-six (36) cases of stump appendicitis by Liang et al. [3] revealed that only 34% of cases were initially performed laparoscopically, and 66% were initially performed as open surgeries, thereby, supporting that it can occur after either laparoscopic or open appendectomy [19].

Stump appendicitis is a real entity not often considered when evaluating patients with right lower quadrant abdominal pain after appendectomy and may be probably an underreported problem [20, 21].

It can occur from about two (2) weeks to an interval of twenty-three (23) year after appendectomy but our case presented four and half (4(1/2)) months postlaparoscopic appendicectomy [14, 15].

Preoperative stump appendicitis diagnosis is still clinical because typically patients present with signs and symptoms similar to acute appendicitis.

Clinicians should have a high index of suspicion for stump appendicitis [22, 23] in patients with a history of previous appendectomy who presented with an acute appendicitis-like picture [24, 25].

Plain films, USD, and CT [26] may all play a role in its diagnosis especially in those associated with abscess formation or perforated cases with intraperitoneal fluid collection in the right lower abdomen or in the pelvis.

The surgical error commonly ascribed to either technique of open or laparoscopic method is the inability in not adequately identifying the base of the appendix, thereby resulting in failure to completely remove [21] the appendix during the initial operation of appendicetomy.

Some authors have suggested stump inversion routinely in all cases after removal of the appendix as a way of minimizing the incidence of stump appendicitis, but others think this is not necessary as long an appendiceal stump of not more than 3 mm in depth is left behind [27, 28].

Different methods of dealing with stump appendicitis include reappendectomy with or without stump inversion, or even limited right hemicolectomy [21, 29, 30].

In our case during exploratory laparotomy, the appendiceal stump with the left behind faecolith was ligated, its base sutured, and the accompanying abscess was drained, followed by peritoneal toilet, abdominal closure and a drain left in situ.

We, therefore, recommend early recognition of this clinical entity to decrease morbidity and high rate of perforation associated with delayed diagnosis [7, 18, 31, 32].

It has therefore been proposed that diagnosis of stump appendicitis should be borne in mind in the differential diagnosis of patients presented with right lower abdominal pain with past history of appendectomy.
